# Supplementary figures and images for: Reward systems for cohort data sharing: An interview study with funding agencies
Source: PLoS One. 2023 Mar 24;18(3):e0282969. doi: 10.1371/journal.pone.0282969 (PMC10038295; doi:10.1371/journal.pone.0282969)

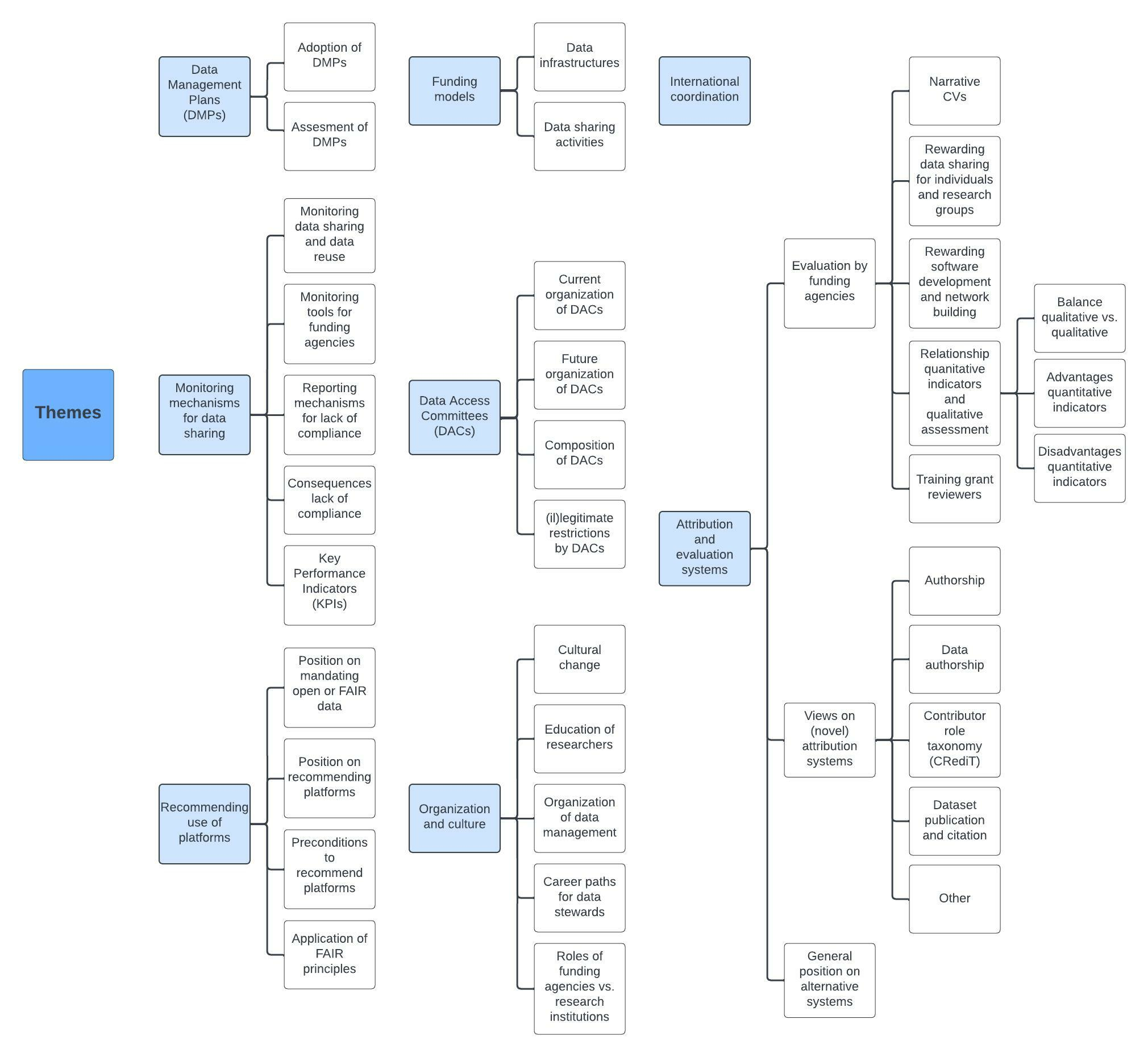

Supplement: S1 Fig — (TIF) [file pone.0282969.s001.tif]
