# Supplementary material for: Reward systems for cohort data sharing: An interview study with funding agencies
Source: PLoS One. 2023 Mar 24;18(3):e0282969. doi: 10.1371/journal.pone.0282969 (PMC10038295; doi:10.1371/journal.pone.0282969)
Supplement: S1 File — (DOCX) [file pone.0282969.s002.docx]

# Terminology

**Attribution system:** Processes of associating performed labor with those responsible for that labor on research outputs

**Evaluation system:** Processes of assessing whether performed labor on outputs is considered valuable in function of particular purposes (e.g., promotion, grant application…)

**Funding distribution system:** Processes of how valuable activities influence the provision of funding to grant applicants

**Recognition system:** Attribution + evaluation system

**Normative criteria:** Value judgement of “credit-worthy” labor that is inherent to authorship models (e.g., in terms of the intellectual nature or types of contributions)

**Reward system:** Processes of how academics are rewarded overall for their activities (e.g., financially, reputational, in-kind…)
